# Three-Dimensional Printing Parameter Optimization for Salmon Gelatin Gels Using Artificial Neural Networks and Response Surface Methodology: Influence on Physicochemical and Digestibility Properties

**DOI:** 10.3390/gels9090766

**Published:** 2023-09-20

**Authors:** Nailín Carvajal-Mena, Gipsy Tabilo-Munizaga, Marleny D. A. Saldaña, Mario Pérez-Won, Carolina Herrera-Lavados, Roberto Lemus-Mondaca, Luis Moreno-Osorio

**Affiliations:** 1 Department of Food Engineering, Universidad del Bío-Bío, Avenida Andrés Bello 720, Chillán 3780000, Chile; nailincm@gmail.com (N.C.-M.); mperez@ubiobio.cl (M.P.-W.); carolina.phl@gmail.com (C.H.-L.); 2 Department of Agricultural, Food and Nutritional Science, University of Alberta, Edmonton, AB T6G 2P5, Canada; marleny.saldana@ualberta.ca; 3 Department of Food Science and Chemical Technology, Universidad de Chile, Santos Dumont 964, Santiago 8330015, Chile; rlemus@uchile.cl; 4 Department of Basic Sciences, Universidad del Bío-Bío, Avenida Andrés Bello 720, Chillán 3780000, Chile; lmoreno@ubiobio.cl

**Keywords:** salmon gelatin, 3D printing, dimensional stability, artificial neural network, secondary structure, digestibility

## Abstract

This study aimed to optimize the 3D printing parameters of salmon gelatin gels (SGG) using artificial neural networks with the genetic algorithm (ANN-GA) and response surface methodology (RSM). In addition, the influence of the optimal parameters obtained using the two different methodologies was evaluated for the physicochemical and digestibility properties of the printed SGG (PSGG). The ANN-GA had a better fit (R^2^ = 99.98%) with the experimental conditions of the 3D printing process than the RSM (R^2^ = 93.99%). The extrusion speed was the most influential parameter according to both methodologies. The optimal values of the printing parameters for the SGG were 0.70 mm for the nozzle diameter, 0.5 mm for the nozzle height, and 24 mm/s for the extrusion speed. Gel thermal properties showed that the optimal 3D printing conditions affected denaturation temperature and enthalpy, improving digestibility from 46.93% (SGG) to 51.52% (PSGG). The secondary gel structures showed that the β-turn structure was the most resistant to enzymatic hydrolysis, while the intermolecular β-sheet was the most labile. This study validated two optimization methodologies to achieve optimal 3D printing parameters of salmon gelatin gels, with improved physicochemical and digestibility properties for use as transporters to incorporate high value nutrients to the body.

## 1. Introduction

Consumers are increasingly interested in ingesting proteins from alternative sources such as plant proteins, insects, algae, and food byproducts [1,2]. A recent example used a byproduct from the fish industry to obtain proteins that have high nutritional and biological value, such as gelatin extracted from fish skin [3]. Gelatin is a coiled, partially hydrolyzed form of collagen of great value for the pharmaceutical, cosmetic, and food industries due to its versatility as an enhancer in a wide range of techno-functional properties. In addition, gelatin is a hydrocolloid that solidifies when it is cold, and it is thermo-reversible, which enables it to exist as a solution or gel, depending on the temperature it is exposed to. Earlier, Carvajal-Mena et al. [3] demonstrated the potential use of salmon skin gelatin as a raw material for 3D printing.

Three-dimensional printing is a novel technology that can develop customized food products with personalized nutrition, and it can create food products easily, economically, and quickly [4]. Furthermore, 3D printing allows the use and combination of various ingredients, including drug administration; thus, adapting food for children, the elderly, and other individuals with special dietary needs [4,5]. However, there are still many reservations about how 3D-printed foods are perceived by consumers. Some studies have shown that repeated consumption of 3D-printed foods can increase acceptability, although in recent times, it has also been claimed that consumers have developed awareness of the health and environmental benefits of these foods compared to conventional production techniques [6]. In the meantime, even though the development and knowledge of this technology are increasing, it is necessary to promote the dissemination of the nutritional advantages and health benefits that 3D-printed foods can offer.

Extrusion 3D printing is the most widely used in the food industry as it can print food inks by extrusion; thus, producing objects with high quality and precision [4,5,6,7]. The development of edible food inks used for the 3D printing process are currently limited. Therefore, optimization of the printing properties and parameters of these food materials is essential to achieve a customized, self-supporting matrix that guarantees smooth extrusion through the nozzle, followed by rapid resolidification to maintain the shape of the printed object [8]. Therefore, it is necessary to optimize the printing parameters for a successful 3D food printing process. Among the important mechanical parameters are the extrusion speed and nozzle diameter and height [9]. The extrusion speed directly affects the stability and quality of the printed geometry, and it has been reported in several studies that a successful process should use speed values between 10 and 70 mm/s [10]. Similarly, nozzle diameter and height influence the volume of the printed object, which is associated with the ability to support weight during printing and prevent deformed printed matrices caused by low printing efficiency. Various studies have suggested that successful printing requires a nozzle diameter between 0.41 and 2.00 mm and a height between 0.3 and 1.1 mm [9,10,11]. Based on this information, it is feasible to establish a methodology to predict matrix printability by optimizing the parameters of the 3D printing process.

Recently, artificial intelligence (AI) has been used in the food industry to optimize extraction processes [12] or to determine specific drying or dehydration conditions [1]. Among AI methodologies, response surface methodology (RSM) is a multivariate strategy that can address both experimental design and statistical modeling by investigating the associations between one or more response variables and a set of experimental factors [12,13]. Therefore, a more efficient method for solving complicated nonlinear problems with significantly high predictive ability is artificial neural network (ANN) modeling, which has been used to establish a more in-depth study of the food industry processes [14]. The ANN is a theoretical mathematical model that emulates the human brain and consists of linear or nonlinear processing elements. The ANN can map complex dynamic phenomena based on self-learning, fault tolerance, and neuron robustness. Meanwhile, genetic algorithms (GA) are a faster optimization technique than traditional algorithms that include a functional probabilistic global search system [15]. Some current research has applied AI to study extrusion processes and its use for predicting physical properties (color and texture) in an extruded rice paste [16] and optimizing 3D printing of chicken meat and the feasibility of the printed product [15]. Furthermore, modeling of the extrusion process of wheat and soybean flour pastes has revealed that the ANN methodology was better adapted to the experimental conditions of the extrusion process than the RSM [17].

Although the experimental optimization of several protein-rich 3D printed foods formulations has been performed, there is no study available that combines 3D printing and AI to design foods with specialized nutritional requirements [1]. In addition, there are no studies on applying these methodologies in 3D printing of protein foods to optimize and predict printing parameters that improve physicochemical properties and increase the digestibility of printed products [18]. Therefore, the main objective of this study was to optimize the 3D printing parameters of salmon gelatin gels (SGG) using artificial neural networks with a genetic algorithm (ANN-GA) and response surface methodology (RSM). In addition, the influence of the optimal parameters obtained using these two different methodologies was determined for the physicochemical and digestibility properties of the printed SGG (PSGG).

## 2. Results and Discussion

### 2.1. Optimization Using Response Surface Methodology

**Figure 1:**
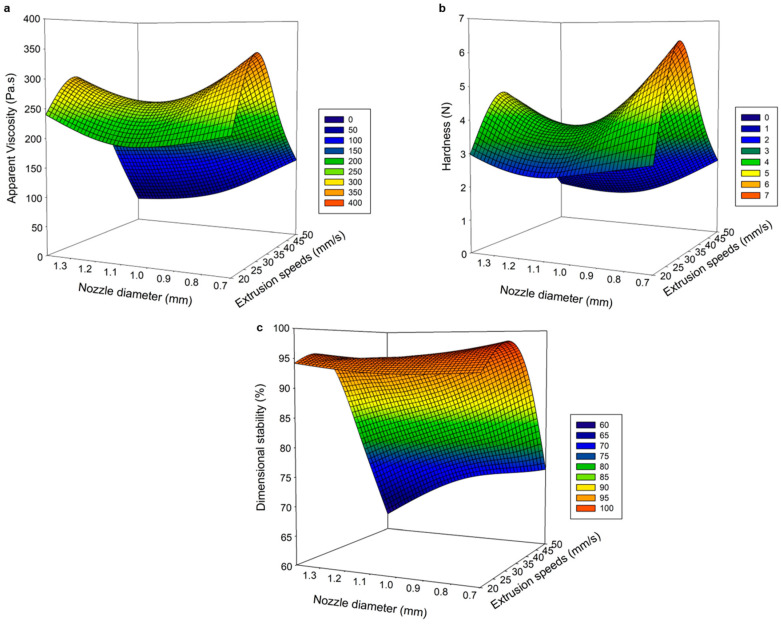
Response surface analysis to optimize salmon gelatin printing parameters related to extrusion speed and nozzle diameter at optimal values for nozzle height. (**a**) Apparent viscosity, (**b**) hardness, and (**c**) dimensional stability.

The ANOVA results enabled the evaluation of the statistical significance of the independent variables in the predicted quadratic model (Table 1). The regression coefficients (R^2^) were 90.61, 87.43, and 96.76% for viscosity, hardness, and dimensional stability, respectively. These regression coefficient values showed that the regression model adequately defined the behavior of the printing variables. The most influential parameters in the printing process were extrusion speed and nozzle diameter because they significantly affected the three response variables to achieve an adequate printed gel. Figure 1 shows the influence of the printing parameters on the physicochemical properties (viscosity, hardness, and dimensional stability) of the 3D PSGG.

Linear (β_1_, β_2_, β_3_), quadratic (β_11_, β_22_, β_33_), and interaction coefficients (β_12_, β_13_, β_23_); intercept (β_0_); and R^2^: coefficient of determination. The nozzle diameter used during the printing process is critical for a high precision surface and finish on the printed gels [19]. Selecting the appropriate nozzle size depends on the printing material and determines the pressure required to achieve gel creep [11]. Figure 1 shows that increasing the nozzle diameter negatively affects the physical properties of the printed gels. In addition, the material flowed faster with larger nozzle sizes and produced thicker lines with poor and rough surfaces (Figure 1). When comparing the three nozzle sizes, the samples printed with the 0.70 mm nozzle diameter exhibited a better appearance that was similar to the designed object, including high dimensional stability and high viscosity and hardness.

The height between the nozzle tip and the printing bed is a fundamental parameter for good quality of the printed object, and a critical height is established for each printing material. In this study, the nozzle height significantly influenced the viscosity of the printed gels. At a lower nozzle height, results showed that the extruded lines were thicker, and lumps formed on the surface of the printed object because the tip of the nozzle was submerged in the material, dragging it along with the movement, and producing deformed and unstable objects. However, when the height was excessive, the gel was deposited in an imprecise manner with irregular lines. Maintaining an adequate nozzle height is required to achieve a well-defined target geometry [19] because the distance from the printed layer to the nozzle tip significantly affects the resolution of printed gels.

The extrusion speed positively affected the physical properties (viscosity, hardness, and dimensional stability) of the printed gels, as it is directly related to the quality and thickness of the extruded filament. As extrusion speed increased, more material was extruded up to a critical point at which the flow was so high that gels were deformed and unstable. Likewise, a low extrusion speed produced a discontinuous extruded filament, which resulted in insufficient structural integrity with a deposit of material that did not cover the nozzle’s trajectory, showing irregular printed gels with broken internal filaments, and an erroneous arrangement of the SGG. Earlier, Southerland et al. [20] reported that an accurate chocolate print was obtained at a slow printing speed but failed to eliminate small air blockages that can occur during extrusion in the syringe. However, Derossi et al. [21] demonstrated that high flow rates were required to achieve better definition and uniformity in the printed object because fruit formulations at low speeds exhibited irregular shapes with discontinuous material lines and unwanted pores. Therefore, extruded lines were thinner and continuous at an optimal extrusion speed; thus, forming high-quality PSGG.

**Figure 2:**
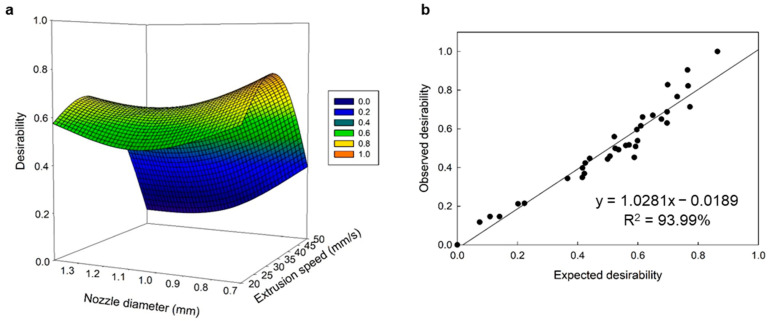
Multiple response optimization analysis. (**a**) Desirability response surface, and (**b**) linear regression analysis between observed and predicted desirability model.

A multivariate optimization was performed to establish the optimal printing parameters for salmon gelatin (Figure 2a). The multiple regression model explained 86.95% of the variability experienced in the response variables. The optimal printing parameters were 31.54 mm/s for the extrusion speed, 0.82 mm for the nozzle diameter, and 0.47 mm for the nozzle height, and the response values were 323.81 Pa·s for the viscosity, 6.43 N for hardness, and 97.78% for the dimensional stability. The linear correlation between the desired optimal and experimental values in the response variables was significant, and the linear model explained 93.99% of the relationship between both variables (Figure 2b).

This study shows how printing parameters significantly influenced the physical properties of the printed gels because, at high extrusion speeds and higher printing nozzle diameters and heights, gels with irregularly shaped extruded filaments and poor definition were formed; thus, leading to negative values of viscosity, hardness, and dimensional stability.

### 2.2. Optimization Using Artificial Neural Networks with Genetic Algorithm

**Figure 3:**
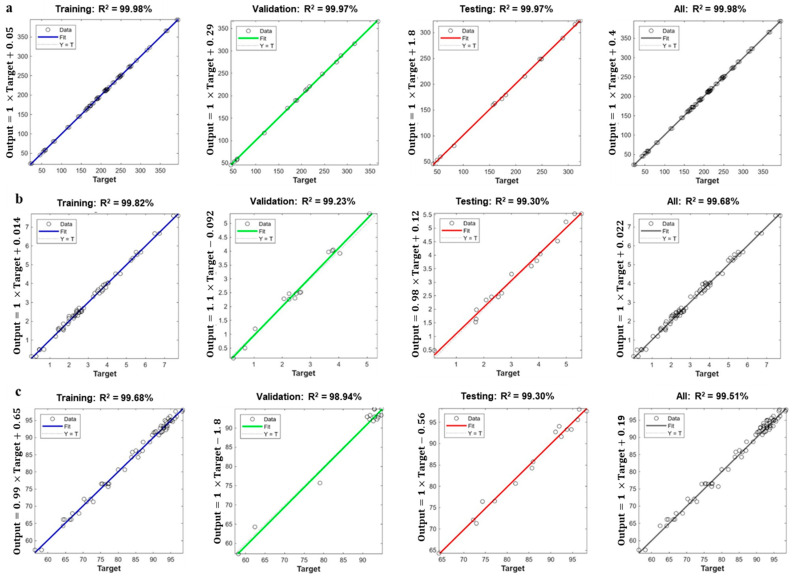
Regression analysis of training, validation, and testing for all the experiments of: (**a**) apparent viscosity, (**b**) hardness, and (**c**) dimensional stability. R^2^ is the coefficient of determination.

The ANN-GA is a modern modeling technique for process optimization because it has high prediction and estimation reliability [21]. In this study, an ANN was trained with a multilayer feed-forward propagation learning algorithm to obtain a function that was optimized by the GA using the variables of the printing process (extrusion speed, nozzle diameter, and height) to maximize the viscosity, hardness, and dimensional stability of the 3D printed SGG (Figure 3). Errors and accuracy of the neural network prediction were performed through multiple rounds of the learning process. In addition, network performance was validated and evaluated by the RSME and R^2^ values obtained at different stages of this study. The R^2^ coefficients for viscosity, hardness, and dimensional stability were 99.88, 99.81, and 99.68% for training; 99.97, 99.29, and 98.94% for validation; and 99.97, 99.29, and 99.30% for testing, respectively. Similar values between training and testing corroborated the accuracy of the ANN model.

Table 2 shows the mean values for sensitivity when evaluating the predictive ability of the input neurons. Results indicated that the most significant predictor was the extrusion speed (100%), followed by the nozzle diameter (55.10%), and nozzle height (30.44%). Furthermore, extrusion speed was the most influential factor on the response variables, which closely concurred with the RSM results. Several studies have indicated that printing speed is a crucial parameter because it directly influences printing efficiency [9]. A lower resolution with unstable structures at faster printing speeds due to the formation of irregular layers was reported, while the printing process slowed down at lower speeds, which also generated structural instability [11,12,13,14,15,16,17,18,19,20,21]. Moreover, nozzle diameter was the second most important parameter where the printing became larger at greater diameters, which led to a poor weight distribution that destabilized the base of the printed gel and caused the figure to collapse. The main drawback of the ANN-GA methodology is that it does not consider the interactions between the different independent variables. However, the accuracy and correlation of the predicted and experimental results were higher with this methodology because it has the potential to train and learn from the experimental data of the response variables.

**Figure 4:**
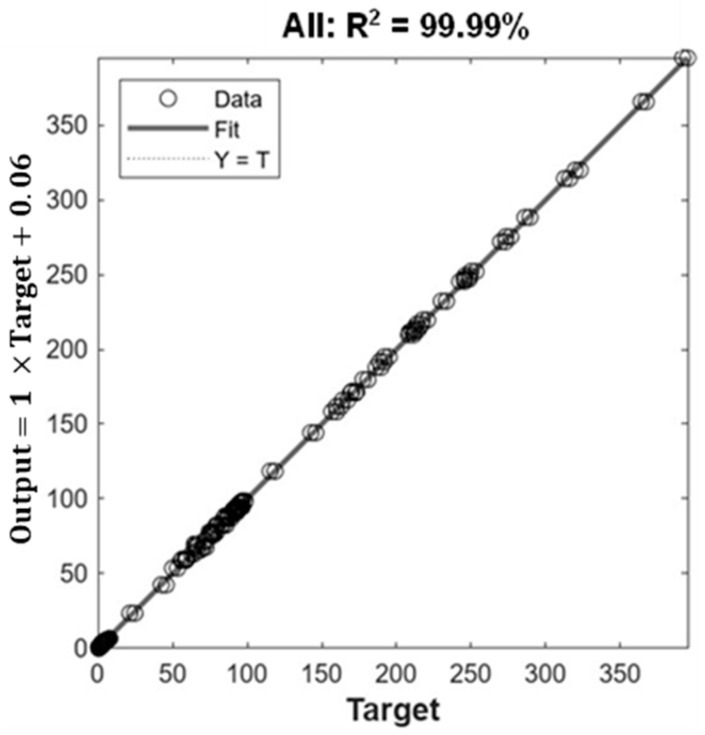
Regression analysis of all the experiments for the total desirability study. R^2^ is the coefficient of determination.

The optimal printing conditions were 24 mm/s for the extrusion speed, 0.70 mm for the nozzle diameter, and 0.50 mm for the nozzle height to achieve 380.85 Pa·s for viscosity, 7.75 N for hardness, and 98.87% for dimensional stability. These predicted values were not significantly different (*p* < 0.05) from the experimental data (R^2^ = 99.98%) under the optimal printing conditions (Figure 4).

### 2.3. Comparison between Response Surface Methodology and Artificial Neural Networks with Genetic Algorithm

The comparison between the RSM and ANN-GA methodologies for predictive ability and statistical accuracy was based on the statistical error parameters. The R^2^ values were higher using the ANN-GA model, indicating that this methodology is more accurate, convenient, and reliable than the RSM for predicting the viscosity, hardness, and dimensional stability of the PSGG. The ANN-GA provided better optimization competence because it learned and predicted models more accurately. In addition, the ANN-GA can also perform limited experiments to process nonlinear relationships between network inputs and outputs and make high-quality predictions.

Several studies have reported the predictive efficiency of the ANN-GA model versus the RSM and have reported its high predictive ability and accuracy [22,23,24]. The validation and testing of the predictive ability of both models involved the predicted values of the models and the experimental data for the different response variables (Figure 1 and Figure 3). The R^2^ values for the RSM and ANN-GA were 93.99% and 99.98%, respectively, which corroborated a better performance of the ANN-GA model. Based on the results obtained, the ANN-GA model exhibited lower residuals with minor variation. In contrast, the deviations between the predicted and experimental responses were more significant in the RSM model.

Although the ANN-GA for the PSGG showed higher quality, stability, and definition with respect to the designed object, the RSM for the PSGG also reported high-correlation values with stable printed structures and no compressed definition of the layers. Therefore, we studied the physicochemical and digestibility properties of the printed gels under the optimal conditions determined using both methodologies.

### 2.4. Thermal Properties

**Figure 5:**
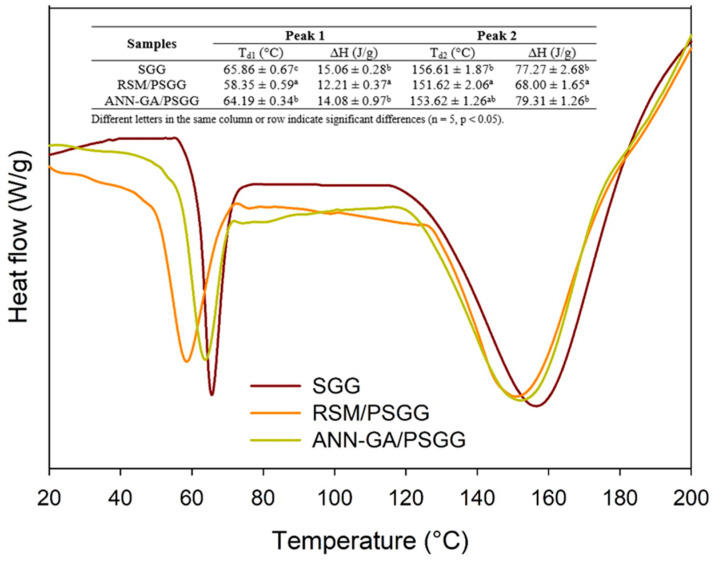
Differential scanning calorimetry thermograms of salmon gelatin gels before and after printing. SGG: salmon gelatin gel; PSGG: printed salmon gelatin gel; RSM: response surface methodology; ANN-GA: artificial neural networks with genetic algorithm.

Figure 5 shows the thermograms obtained using the DSC analysis of the gels before printing and after printing at optimal conditions using the RSM and ANN-GA methods. All the gels had two peaks of thermal transitions: the first peak corresponded to the glass transition and the second peak corresponded to the protein denaturation (Figure 5).

The glass transition temperatures (Tg) varied between 58.35 and 65.86 °C, and the Tg values of the gel before printing were significantly higher than the printed gels under both optimal conditions. This higher transition temperature in the SGG occurred due to the larger number of bonds and interactions between the gelatin chains that promoted thermal stability [25]. In addition, gelatin tends to absorb water from the medium, which means that evaporation of this crystallizable water is also responsible for an increase in Tg [25].

Meanwhile, the pressure exerted on the gels during the extrusion process also affects the Tg values. The ANN-GA for the PSGG gels had higher Tg values than the RSM for PSGG gels that can be associated with lower free volume and higher water loss during extrusion 3D printing. In addition, the ∆H values of SGG and ANN-GA/PSGG showed no significant differences but were significantly higher than the RSM/PSGG values. The smaller nozzle diameter used for printing the ANN-GA/PSGG gels generated higher pressure to which they were subjected; thus, causing minimal polymer expansion and creating more compact, crosslinked molecular chains, which required higher energy to achieve molecular mobility [26].

The denaturation temperature and enthalpy of the SGG were slightly higher than for the PSGG due to the more compact molecular bonds that required higher temperature and energy to achieve the helix–spiral transition of the gelatin and break the associated hydrogen bonds.

### 2.5. In Vitro Digestibility

**Figure 6:**
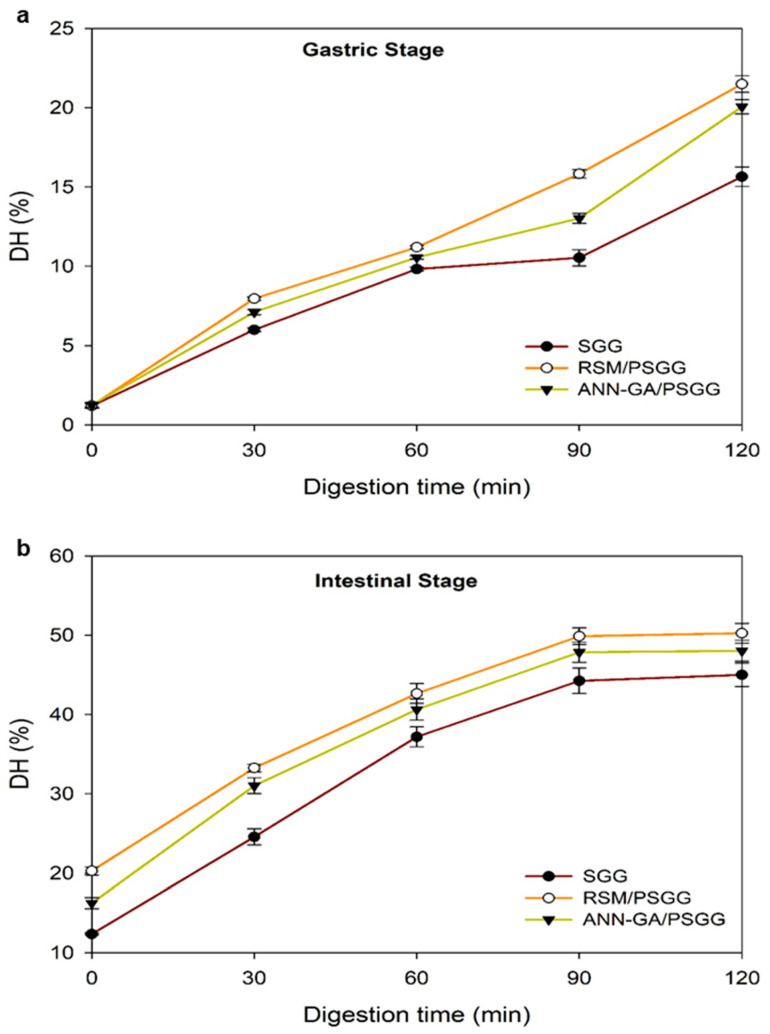
Degree of hydrolysis of gelatin gels at: (**a**) gastric stage, and (**b**) intestinal stage. DH: degree of hydrolysis; SGG: salmon gelatin gel; PSGG: printed salmon gelatin gel; RSM: response surface methodology; ANN-GA: artificial neural networks with genetic algorithm.

Figure 6 shows the digestibility of the SGG and PSGG. The DH increases slightly during the gastric stage, while its increase in the intestinal stage was significant in the first 30 min for all gels studied. Then, pancreatic enzymes broke peptide bonds during the intestinal stage, which created more active sites for further hydrolysis [27]. During the gastric stage, the DH was 15.64% for the SGG and 20.06–21.49% for the PSGG, showing significant differences between the gels before and after printing under the different conditions.

The DH value of the SGG in the intestinal stage was 46.93%, with significant differences between gels printed under both conditions, indicating that the extrusion 3D printing process significantly affected the DH. The protein extrusion process caused conformational changes such as unfolding, association, aggregation, and crosslinking of molecular chains for protein degradation or oxidation [28]. In addition, the extrusion process was an efficient way to increase digestibility due to breaks in the polymeric chains that comprise the protein structure. Although there were no significant differences between both printed gels, the DH values for the RSM/PSGG were higher than for the ANN-GA/PSGG during the whole digestive process. Therefore, during gel formation under the optimal conditions provided by the RSM, gel network formation was altered, which left more empty spaces between the layers; thus, enabling the enzymes to enter and diffuse more easily.

The DH for both the SGG and PSGG were higher than values reported in the literature for other gelatins of marine origin. Earlier, Wang et al. [27] reported values of 31.79% for gelatin from Nile tilapia flakes. Overall, the results of the gastrointestinal digestion analysis showed how the extrusion 3D printing process improved the digestibility of salmon gelatin by increasing the number of bioaccessible amino acids (glycine and proline) for subsequent gastrointestinal absorption.

### 2.6. Fourier Transform Infrared Spectroscopy with Attenuated Total Reflectance

**Figure 7:**
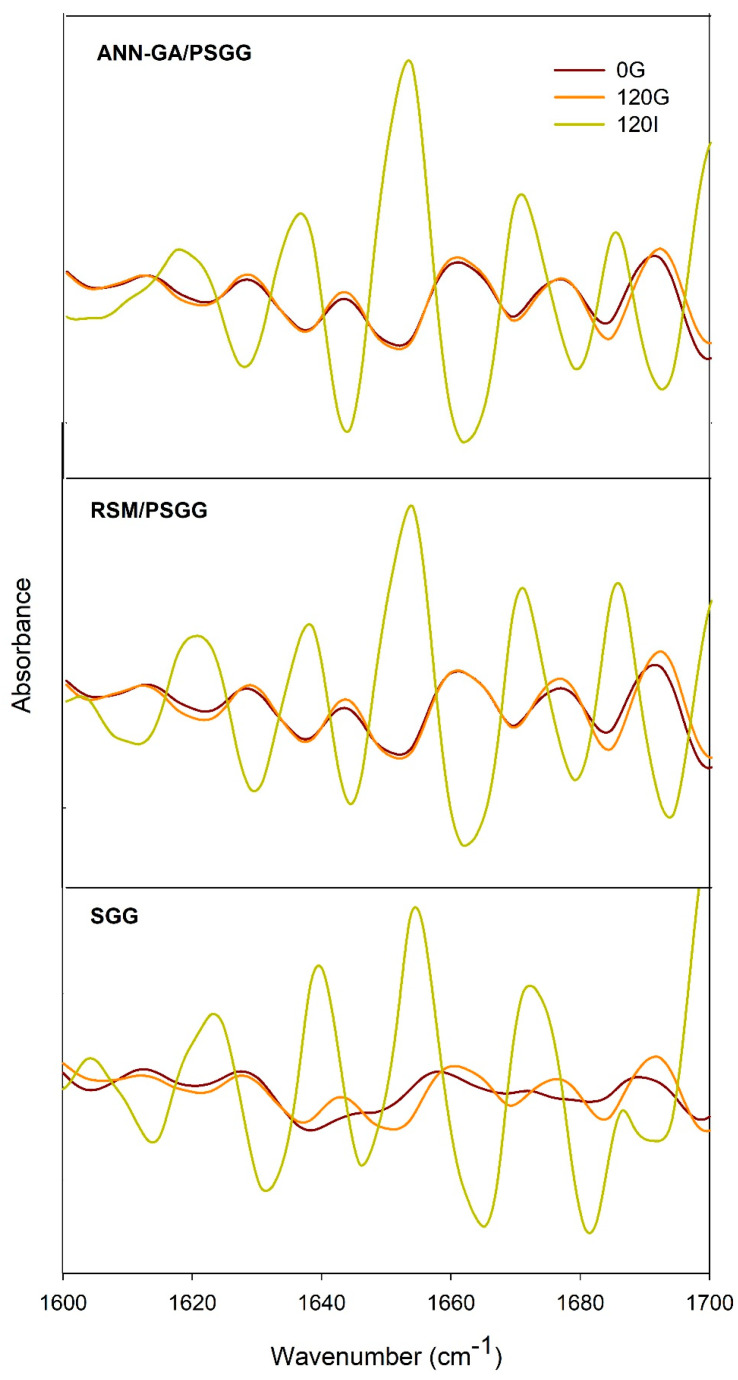
Gelatin secondary structure determined using Fourier transform infrared spectroscopy (FTIR). SGG: salmon gelatin gel; PSGG: printed salmon gelatin gel; RSM: response surface methodology; ANN-GA: artificial neural networks with genetic algorithm.

The secondary structures of the SGG and PSGG gels printed under optimal conditions during the digestion process were analyzed by studying the area of the bands in the amide I region (1600–1700 cm^−1^), which consisted of C=O bond stretching vibrations and, to a lesser extent, C-N stretching vibrations in the polypeptide chain (Figure 7). The quantitative analysis of the second derivative spectra showed that all the gels exhibited six structures in the amide I region: 1604–1623 cm^−1^ β-sheet intermolecular; 1641–1645 cm^−1^ random coil; 1650–1654 cm^−1^ α-helix; 1661–1668 cm^−1^ β-turn; 1681–1684 cm^−1^ intramolecular antiparallel β-sheet; and 1699–1701 cm^−1^ antiparallel β-sheet [29]. The predominant protein structure in all the gels was the antiparallel β-sheet with initial values between 35.08% and 37.49%. Meanwhile, the α-helix structure had values close to 20%, similar values to findings reported by Yang et al. [29] for other gelatins of marine origin (Salmon and Alaska pollock).

**Figure 8:**
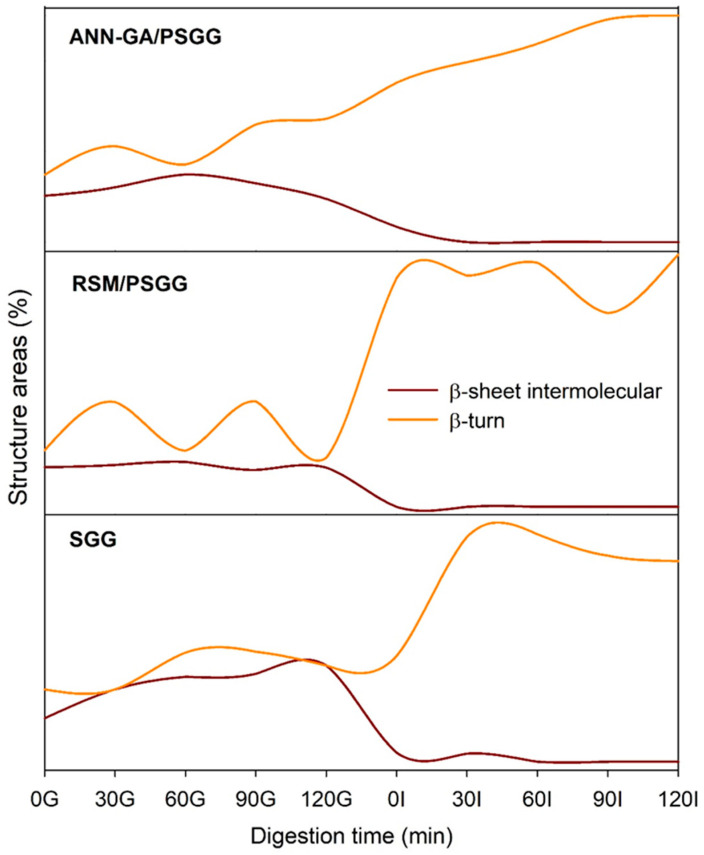
Percentage of the most resistant and most labile areas of the secondary structure of salmon gelatin gels during the digestive process. SGG: salmon gelatin gel; PSGG: printed salmon gelatin gel; RSM: response surface methodology; ANN-GA: artificial neural networks with genetic algorithm.

The variations that occurred in the secondary structures of the gelatin gels during the digestion process showed that the β-turn structure increased significantly and was the most resistant to enzymatic hydrolysis of the structures in the gels (Figure 8). The intermolecular β-sheet structures decreased significantly as the DH increased, reaching zero during the intestinal digestion stage, which demonstrated that this structure is the most labile. The behavior of the structure was directly correlated with the higher DH shown by the printed gels, which indicated that greater protein denaturation occurred due to the action of pancreatic enzymes during the intestinal stage. After completing the digestive process, all the gels still exhibited bands of various protein structures, which could be related to the remaining collagen protein that was not hydrolyzed during the digestive process.

## 3. Conclusions

The ANN-GA methodology correlated better with the experimental conditions of the 3D printing process than with the RSM methodology. Among the three printing parameters evaluated, the extrusion speed was the most influential parameter established by both methodologies. The optimal printing parameter values established by the ANN-GA methodology were 0.70 mm for the nozzle diameter, 0.5 mm for the nozzle height, and 24 mm/s for the extrusion speed. The final printed product values were 380.85 Pa·s for the apparent viscosity, 7.75 N for hardness, and 98.87% for the dimensional stability, and there was a 99.98% correlation between predicted and experimental values. The optimization methodologies directly influenced the quality of the printed gels by affecting the thermal properties because more compact structures were formed, which required higher denaturation energy and temperatures. The extrusion 3D printing process improved the digestibility of salmon gels with values ranging from 46.93% (SGG) to 51.52% (PSGG). In addition, the gel’s secondary structure was modified during the digestive process that caused higher hydrolysis of the intermolecular β-sheet structures, which were the most labile of all the gels investigated, while the β-turn structure significantly increased and was the most resistant. This study demonstrates that artificial intelligence can be used to predict 3D printing process variables of food matrices to produce high-quality printed objects with improved physicochemical and digestibility properties that can be used as a transporter for incorporating high value nutrients to the body.

## 4. Materials and Methods

### 4.1. Materials

Salmon gelatin was extracted from coho salmon (Oncorhynchus kisutch) skins obtained from the Salmones Aysén S.A. salmon processing plant (Puerto Montt, Chile). The skins were washed with water to remove muscle debris and scales, cut into squares (20 mm), and stored at −20 °C until further use. All reagents and enzymes were purchased from Sigma–Aldrich Co. (St. Louis, MO, USA).

### 4.2. Salmon Gelatin Extraction

Salmon gelatin was extracted following the methodology reported by Carvajal-Mena et al. [3]. First, salmon skins (100 g) were immersed in a sodium hydroxide solution (2 g/L) in a 1:3 (*w*/*v*) ratio and shaken at a constant speed of 1200 rpm for 1 h. Then, they were washed 3 times in 1 L of distilled water to remove all traces of sodium hydroxide. They were treated in an acetic acid solution (4.2 g/L) at a skin/solution ratio of 1:3 (*w*/*v*) with constant agitation at 1200 rpm for 1 h and washed 3 times with distilled water (1 L) to eliminate any acetic acid in the samples. The gelatin extraction process was conducted at 45 °C for 24 h. The supernatant liquid was filtered, and the gelatin was removed. The supernatant liquid was vacuum filtered with a paper filter (Whatman 22 mm, Merck, Darmstadt, Germany) and frozen at −80 °C for freeze-drying in a laboratory freeze dryer (model Beta 1–8 LD, Martin Christ Gefriertrocknungsanlagen, Osterode am Harz, Germany) that was maintained at −42 °C and 10.21 Pa pressure for 24 h. The freeze-dried gelatin was stored at 4 °C until further use.

### 4.3. Preparation of Salmon Gelatin Gels

Based on preliminary results by Carvajal-Mena et al. [3], it was established that an 8% gelatin concentration was needed to obtain a stable salmon gelatin gel (SGG) for the 3D printing process. First, salmon gelatin (8 g) was dissolved in distilled water (100 mL) at 60 °C for 30 min, and the glass container was covered with aluminum foil during dissolution to prevent water loss. The resulting solution was cooled to room temperature and stored at 6 °C until further printing.

### 4.4. Dimensional Printing

**Figure 9:**
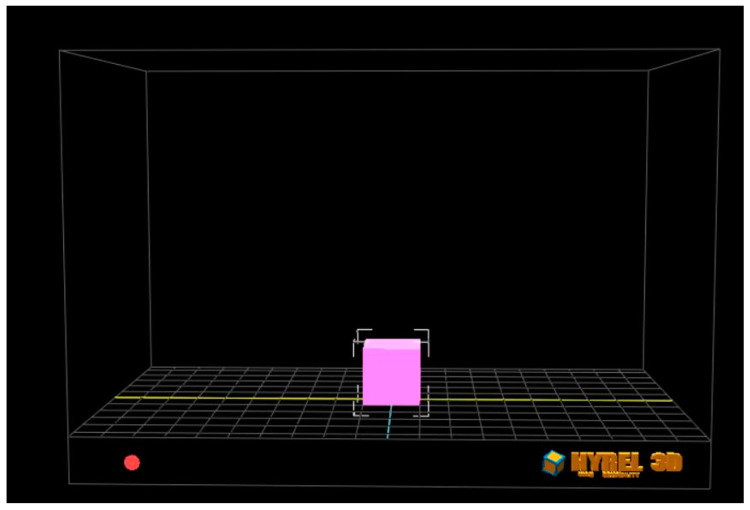
Image of the object to be printed on the Repetrel software platform. The pink cube shows the design of the figure to be printed, represented in a 3D coordinate system that simulates the printing platform. The blue and yellow lines mark the center of the platform on the “x” and “y” axes, respectively.

The SGG was printed with a 3D System 60M printer (Hyrel International Inc., Norcross, GA, USA). Stereolithography files with cubic designs (Figure 9; 20 mm width and length, and 30 mm height) were converted to G-codes with the Repretel software (Hyrel International Inc., Norcross, GA, USA). The printing process was optimized by studying the nozzle height and diameter and extrusion speed variables. The SGG were printed at four extrusion speeds (20, 30, 40 and 50 mm/s); three nozzle diameters (0.7, 1.05 and 1.4 mm); and three nozzle heights (0.3, 0.5 and 0.7 mm). The printing temperature (15 °C) and printing bed temperature (6 °C) were controlled throughout the process using Repretel software version 3.083.

### 4.5. Optimization of 3D Printing Process

#### 4.5.1. Response Surface Methodology

This study used a multifactorial design with three main variables. The second-order polynomial model represented viscosity, hardness, and dimensional deviation as a function of three independent variables (nozzle diameter, nozzle height, and extrusion speed). The experiments were randomly conducted, and data analyses were performed with the Statgraphics Centurion XVI statistical software (Statistical Graphics Corp., Herdon, VA, USA). The response surface model describing the behavior of the dependent variables (viscosity, hardness, and dimensional deviation) as a function of the three independent variables is expressed in Equation (1), as follows:

(1) $$Y=\beta_{0}+\sum_{i=1}^{3}\beta_{i}X_{i}+\sum_{i=1}^{3}\beta_{ii}X_{i}^{2}+\sum_{i=1}^{2}\sum_{j=i+1}^{3}\beta_{ij}X_{i}X_{j}$$

where *β*_0_ is the constant; *β_i_*, *β_ii_*, and *β_ij_* are regression coefficients; *X_i_* and *X_j_* are the levels of coefficients that are independent of the variables; and *Y* is the dependent variable.

**Figure 10:**
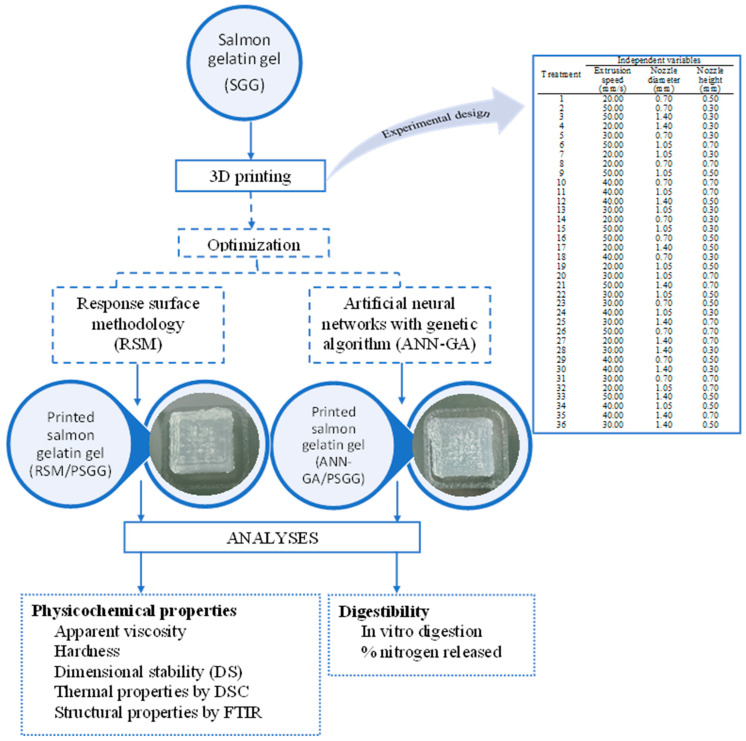
Experimental design for salmon gelatin gel.

This experimental study design consisted of 36 experiments (Figure 10). Experiments were randomized, and averages were used for the analyses to minimize variability in the response variables. The impact of the independent variables on the dependent variables was accounted for using analysis of variance (ANOVA) of the data at *p* < 0.05.

#### 4.5.2. Artificial Neural Networks with Genetic Algorithm

Parameter modeling and optimization of the 3D printing process of SGG were performed by artificial neural networks with the genetic algorithm (ANN-GA) using the MATLAB mathematical software (version 2023a, Natick, MA, USA). The ANN model was constructed by the backpropagation technique and feed-forward neural network. It consisted of different weights and biases between three input neurons for nozzle height, nozzle diameter, and extrusion speed and three output neurons for the viscosity, hardness, and dimensional stability response variables. The multilayer perceptron algorithm in the ANN modeling was trained with the Levenberg–Marquardt algorithm in 108 runs, including the 3 replicates of each of the 36 experiments. The ANN modeling uses replicates instead of mean values to assess model variability and variances. The runs were divided into three parts, 70% training, 15% validation, and 15% test sets. The training data were used to fit the parameters of the network model; the test data were used to calculate the estimation accuracy; and the validation data were used to ensure the robustness of the network and its parameters. The number of neurons in the hidden layers was determined by a maximum R^2^ and a minimum mean square error (MSE) value that were found in 12 hidden neurons in which the transfer function was a hyperbolic sigmoid function for the hidden layer and a linear function for the output layer.

The genetic algorithm (GA) is a multi-objective optimization search technique based on the principles of biological evolution [15]. The ANN model was convolved with GA to optimize the variables of the 3D printing process and maximize the desired output variables. The optimization parameters had a population size of 50 and a cross probability fraction of 0.8.

#### 4.5.3. Model Validation

The models were validated by applying the two statistical parameters, root mean square error (*RMSE*) and coefficient of determination (*R*^2^), using Equations (2) and (3), respectively.

(2) $$RMSE=\sqrt{\frac{\sum_{i=1}^{n}{\left(Y_{ei}-Y_{pi}\right)}^{2}}{n}}$$

(3) $$R^{2}=1-\sum_{i=1}^{n}(\frac{{\left(Y_{ei}-Y_{pi}\right)}^{2}}{{\left(Y_{m}-Y_{pi}\right)}^{2}})$$

where *n* is the number of experiments; *Y_ei_* is the experimental viscosity, hardness, or dimensional stability; *Y_pi_* is the predicted viscosity, hardness, or dimensional stability; and *Y_m_* is the average viscosity, hardness, or dimensional stability.

### 4.6. Apparent Viscosity

The apparent viscosity of the printed SGG (PSGG) under the different printing conditions was determined with a controlled strain and tension rheometer (Physica MCR 300, Anton Paar, Filderstadt, Germany). The instrument was equipped with serrated parallel plate geometry (25 mm diameter) with a 1 mm GAP; the shear rate varied between 0.1 and 100 s^−1^ at a constant temperature of 6 °C. The data of the apparent viscosity curves of the SGG were recorded with the US200 Physica software version 2.01. The viscosity value was determined in the portion of the curve where it became linear.

### 4.7. Hardness

The hardness of the PSGG was determined with a texture analyzer (TAXT plus100, Stable Micro Systems Ltd., Godalming, UK). The SGG underwent two compression cycles with a cylindrical probe (Code P/50, Stable Micro System Ltd., Godalming, UK). Test conditions were 2 mm s^−1^ pre-test velocity, 1 mm s^−1^ test velocity, and 5 mm s^−1^ post-test velocity, while compression was 50% of its original height at 6 ± 2 °C. The time between the first and second compression was 3 s. The hardness was determined by the force–time curve.

### 4.8. Dimensional Stability

Dimensional stability was determined according to the method reported by Carvajal-Mena et al. [3], using a cube (20 mm width and length, and 30 mm height) as a standard shape. The length, width, and height dimensions were measured with a Vernier caliper in five different positions for each direction. Mean values were used, and this measurement was performed after 24 h of printing at 6 ± 2 °C. The dimensional stabilities were determined according to Equations (4) and (5).

(4) $${DS}_{L,W,H}=\frac{\left(Measured\;value-Target\;value\right)}{\;Target\;value\;}$$

(5) $${\%\;DS}_{T}=\frac{{DS}_{L}+{DS}_{W}+{DS}_{H}}{3}\times 100$$

where *DS_T_* is the total dimensional stability and *DS_L_*_,*W*,*H*_ is the dimensional stability for length, width, and height, respectively.

### 4.9. Thermal Properties

The thermal properties of the SGG were analyzed by differential scanning calorimetry (DSC) using a DSC instrument (model DSC1, Mettler Toledo AG, Analytical, Schwerzenbach, Switzerland). Approximately 10 mg of wet gels were placed in a 40 μL airtight aluminum container with distilled water and used as a reference. The gels were first subjected to an isothermal phase at 6 °C for 5 min and then scanned from 20 to 200 °C at a rate of 5 °C/min. Denaturation temperature and enthalpy were estimated by determining the area under the transition curves with the Stareware version 10.01 software (Mettler Toledo, Schwerzenbach, Switzerland).

### 4.10. Dynamic In Vitro Simulation of Human Gastroduodenal Digestion

The digestibility of in-mold and PSGG under the optimal conditions obtained by the RSM and ANN was determined by a dynamic model of in vitro human digestion [30]. First, the gels were subjected to the oral phase, mixed with simulated salivary fluid (1:1 *v*/*v*) to a total volume of 40 mL, and α-amylase was added. Then, the solution was vigorously shaken for 2 min and was immediately afterward placed in a gastric bioreactor (MiniBio, Applikon Biotechnology, Delft, The Netherlands). Subsequently, 60 mL of simulated gastric fluid with pepsin was added to the gastric bioreactor at 37 °C, and CaCl_2_ was incorporated to start the “my-Control” version 1.0X control software (Applikon, Delft, The Netherlands). The software was programmed to perform a gastric pH gradient from 4.5 to 1.2 and intermittent pulses at 200 rpm. The software also controlled the passage of the digested gastric solution to the duodenal bioreactor through a peristaltic pump 10 min after the start of the digestive process. According to Levi and Lesmes [30], 10 mL of simulated duodenal fluid was added to the duodenal bioreactor (MiniBio, Applikon Biotechnology, Delft, The Netherlands), the temperature was maintained at 37 °C, pH was constant at 6.1, and a physiological rate of bile secretion was controlled by a peristaltic pump. The pancreatic enzymes trypsin and α-chymotrypsin were added in two pulses, the first at 10 min and the second at 50 min after the start of the intestinal phase. Total digestion time of the experiment was set at 2 h, and samples were collected from the gastric and duodenal bioreactors at 0, 30, 60, 90 and 120 min. Gastric solutions were neutralized by rapidly raising the pH to 7 with NaOH (1 M), while intestinal solutions were quenched with PMSF (0.5 mM phenylmethylsulfonyl fluoride). Samples were stored at −20 °C until further use.

### 4.11. Degree of Hydrolysis

The degree of hydrolysis (DH) of the protein was determined by the o-phthaldialdehyde (OPA) method described by Nielsen et al. [31]. The OPA reagent was prepared according to the methodology described by Opazo-Navarrete et al. [32]: 160 mg of OPA were dissolved in 4ml ethanol and then were added to a previously prepared solution containing 7.62 g borax, and 200 mg of SDS dissolved on 150 mL deionized water, mixed well and 176 mg of dithiothreitol (DTT) was added, made up to 200 mL and then stored in an amber bottle for 24 h after preparation. The L-serine standard curve was prepared at concentrations ranging from 50 to 200 mg/mL [31]. The digested samples were centrifuged for 20 min at 14,000× *g*, and the supernatant was used for measurements. A total of 200 µL of supernatant was mixed with 1.5 mL of OPA reagent for 3 min, after which absorbance was measured at 340 nm with a spectrophotometer (Spectroquant Pharo 300, Merck KGaA, Darmstadt, Germany). Free amino groups in the digested salmon gelatins were expressed as serine amino equivalents (serine NH2). DH was calculated using Equations (6) and (7).

(6) $$DH=\frac{h}{h_{tot}}\times 100\%$$

(7) $$h=\frac{Serine\;NH_{2}-\beta }{\alpha }$$

where the values of the constants were α = 0.796 and *β* = 0.457 [28]. The *h_tot_* was determined as a function of the concentration of each amino acid in the protein and had a value of 13.25 milliequivalent/g.

### 4.12. Fourier Transform Infrared Spectroscopy with Attenuated Total Reflectance

The Fourier transform infrared spectroscopy (FTIR) spectra of in-mold and PSGG and digests were recorded with an IRPrestige-21 spectrometer (Shimadzu 21 Corporation Pte. Ltd., Kyoto, Japan). Approximately 50 µL of the solution was placed on the attenuated total reflectance (ATR) glass and 128 scans were performed in the absorption mode with a 4.0 cm^−1^ resolution at wavelengths from 4000 to 400 cm^−1^. The amide I region (1600–1700 cm^−1^) was smoothed with a twelve-point Savitsky–Golay function, and the normalized second derivative spectra were obtained with the IRsolution version 1.10 software (Creon Lab Control AG, Shimadzu Corporation Pte. Ltd., Kyoto, Japan). Gaussian curve-fitting was performed on the different second derivative IR spectra with the Origin version 9.0 PRO software (OriginLab Corporation, Northampton, MA, USA). The content of each of the structures was calculated from the area under the relative peaks of the assigned bands.

### 4.13. Statistical Analysis

Results were analyzed with the Statgraphics Centurion XVI statistical software (Statistical Graphics Corp., Herdon, VA, USA). Each experiment was performed in triplicate with three samples in each replicate. Fisher’s LSD test was used to minimize significant differences with a 95% confidence interval.

## Figures and Tables

**Table 1:** Regression coefficients of the second-order polynomial models predicted for the response variables.

Regression Coefficient	Estimated Coefficient		
	Apparent Viscosity	Hardness	Dimensional Stability
β_0_	−149.34	−7.59	35.01
β_1_	36.17	0.70	3.48
β_2_	−535.37	−16.64	11.48
β_3_	758.42	44.17	49.23
β_11_	−0.49	−0.009	−0.05
β_12_	−5.33	−0.045	−0.66
β_13_	−2.09	−0.05	−0.67
β_22_	304.42	7.67	1.18
β_23_	30.35	1.29	−0.21
β_33_	−761.45	−44.96	−35.64
R^2^ (%)	90.61	87.43	96.76

**Table 2:** Artificial neural networks with genetic algorithm (ANN-GA) sensitivity analysis of salmon gelatin gels.

Neural Network (NN)	Extrusion Speed	Nozzle Diameter	Nozzle Height
	(mm/s)	(mm)	(mm)
NN1	0.35	0.24	0.42
NN2	0.24	0.63	0.13
NN3	0.58	0.10	0.33
NN4	0.52	0.44	0.04
NN5	0.88	0.02	0.09
NN6	0.78	0.11	0.11
NN7	0.42	0.35	0.23
NN8	0.60	0.29	0.11
NN9	0.49	0.31	0.20
NN10	0.48	0.46	0.07
NN11	0.60	0.37	0.04
NN12	0.54	0.25	0.20
Average Importance	0.54	0.30	0.16
Normalized Importance (%)	100.00	55.10	30.44

## Data Availability

The data presented in this study are available upon request from the authors.
